# Complete mitochondrial genome of *Rivularia auriculata* (Gastropoda, Viviparidae) with phylogenetic consideration

**DOI:** 10.1080/23802359.2019.1688696

**Published:** 2019-11-13

**Authors:** Chunxia Zhang, Jing Guo, Huirong Yang, Jia-En Zhang

**Affiliations:** a College of Natural Resources and Environment, South China Agricultural University, Guangzhou, PR China;; b Henry Fok College of Life Sciences, Shaoguan University, Shaoguan, PR China;; c College of Marine Sciences, South China Agricultural University, Guangzhou, PR China;; d Guangdong Provincial Key Laboratory of Eco-Circular Agriculture, Guangzhou, PR China;; e Key Laboratory of Agro-Environment in the Tropics, Ministry of Agriculture, Guangzhou, PR China;; f Guangdong Provincial Engineering Technology Research Center of Modern Eco-agriculture and Circular Agriculture, Guangzhou, PR China

**Keywords:** *Rivularia auriculata*, mitochondrial genome, NGS technique

## Abstract

Complete mitochondrial genome sequence of *Rivularia auriculata* has a circular genome of 16,552 bp, which is comprised 13 protein-coding genes, 2 rRNA genes, and 22 tRNA genes. The nucleotide composition of the light strand is 43.16% of A, 26.78% of T, 20.18% of C, and 9.88% of G. All genes are encoded on the heavy strand except seven tRNA genes (*Met*, *Tyr*, *Cys*, *Trp*, *Gln*, *Gly*, and *Glu*) on the light strand. All the protein-coding genes start with ATC initiation codon except ND4 starts with GTG, and two types of inferred termination codons are TAA and TAG. There are 26 intergenic spacers and 4 gene overlaps. It is indicated that *R. auriculata* has closer genetic relationship with *Viviparus chui* (88.64% nucleotide sequence identity between them) than the other snail species.

*Rivularia auriculata* is a kind of rare species of China, which mainly distributes in Xiangjiang River basin of Hunan province, and also scatters in neighboring provinces. The species belongs to the family Viviparidae and the order *Architaenioglossa*. Due to the destruction of its natural habitats and decreasing of its populations, *R. auriculata* was listed as Extinct Species by China Red Data Book of Endangered Animals. This presents a major obstacle for conservation efforts.

So far there are only a few researches on the population dynamics and morphological structure of *R. auriculata* (Zhou 1996; Pan et al. 2010), and no mitochondrial genomes of genus *Rivularia* in NCBI PubMed. Therefore, we sequenced the complete mitochondrial genome of *R. auriculata* by using the next-generation sequencing (NGS) techniques for its further population genetics and polymorphism studies. *R. auriculate* samples were collected from Poyang Lake in Jiangxi province of China (28°74′ N, 116°41′ E). Specimen（voucher no. zcx20181109-10）was preserved in 95% ethanol and stored at −40 °C refrigerator in the Key laboratory of Eco-circular Agriculture in South China Agricultural University, Guangzhou, China. The procedure referred from Green and Sambrook (2012) was carried out in the total genomic DNA extraction.

We sequenced and characterized the complete mitochondrial genome of *R. auriculata*. The mitochondrial DNA sequence of *R. auriculata* with the annotated genes was deposited in GenBank (accession number: MN264502). Complete mitochondrial genome sequence of *R. auriculata* is a circular one of 16,552 bp containing a total of 37 genes, including 13 protein-coding genes, 2 ribosomal RNA genes (12S-rRNA and 16S-rRNA), and 22 transfer RNA genes (tRNA). All of them are encoded on the heavy strand except seven tRNA genes (*Met*, *Tyr*, *Cys*, *Trp*, *Gln*, *Gly*, and *Glu*). The inner ring indicates the GC percent varying from 17.39% to 38.10%. The overall nucleotide composition of the light strand in descending order is 43.16% of A, 26.78% of T, 20.18% of C, and 9.88% of G. The most representative base is A, and the bias against G is observed. Among 13 protein-coding genes (total 11,265 bp) encoding 3742 amino acids, the maximum is ND5 with 1719 bp and the minimum is ATP8 with only 174 bp. The 12S-rRNA and 16S-rRNA genes are 897 and 1361 bp, respectively, located between the tRNA^Glu^ and tRNA^Leu^ genes and separated by the tRNA^Val^ gene. The phenomenon of D-loop absence is consistent with the Gastropoda (Zeng et al. 2015; Yang, Zhang, Deng et al. 2016; Yang, Zhang, Guo, et al. 2016; Yang, Zhang, Luo et al. 2016), but at least one lengthy non-coding region is an essential regulatory element for the initiation of transcription and replication (Wolstenholme 1992).

All protein-coding genes start with ATG initiation codon except ND4 with GTG, and two types of inferred termination codons are TAA (*COX1*, *ATP8*, *ND1*, *ND6*, *CYTB*, *ND4L*, *COX3*, *ND3*, and *ND4*) and TAG (*COX2*, *ATP6*, *ND5*, and *ND2*). Twenty two tRNA genes vary from 60 to 70 bp in length, and all fold into the typical cloverleaf secondary structure. There are 26 intergenic spacers (total 1567 bp) varying from 1 to 946 bp in length and 4 gene overlaps (total 17 bp), the larger of which is 7 bp between the *CYTB* and *ND5* genes. The tandem repeat sequences are observed in inter genetic space of tRNA^Thr^ (TGT).

Phylogenetic analysis covered mitochondrial genomes of *R. auriculata* and the other 14 species that are from the order *Bellamya, Cipangopaludina, Margarya, Rivularia, Viviparus, Pomacea, Marisa,* and *Obscurella,* belong to Architaenioglossa (Figure 1), and *Littorina saxatilis* was used as an outgroup. Maximum likelihood (ML) method was adopted for phylogenetic analysis (Figure 1). The GTR + I+G model was selected by the Akaike information criterion in jModelTest2.1.7 (https://code.google.com/p/jmodeltest2/). The phylogenetic tree was visualized with MEGA version 6.06 (Tamura et al. 2013). It is indicated that *R. auriculata* has closer genetic relationship with *Viviparus chui* (88.64% nucleotide sequence identity between them than the other snail species (Figure 1).

**Figure 1.**
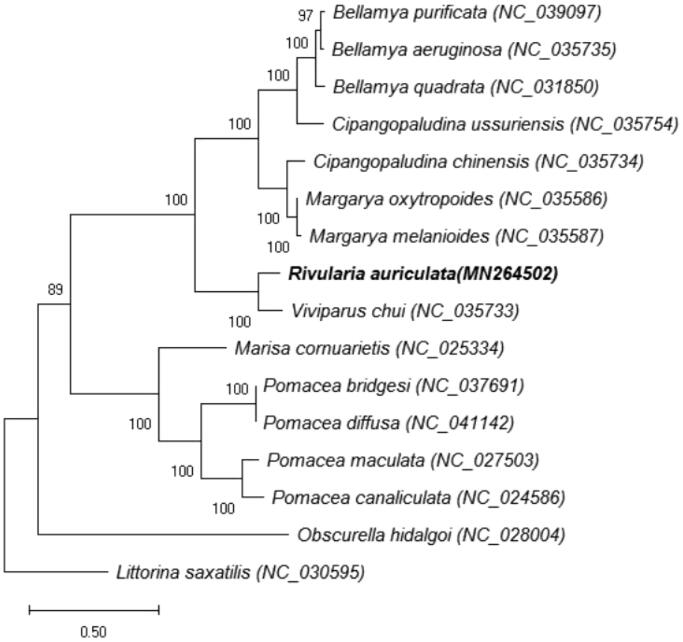
Phylogenetic tree of maximum-likelihood was constructed in RAxML based on the nucleotide sequences of 13 protein-coding genes. The bootstrap values were based on 1000 resamplings.

## References

[CIT0001] Green MR, Sambrook J 2012 Molecular cloning: a laboratory manual. 4th ed. New York (NY): Cold Spring Harbor Laboratory Press.

[CIT0002] Pan H, Ouyang S, Huang P, Zhao D, Ruan L, Wu X 2010 Population dynamics, annual production of *Rivularia auriculate* in Junshan Lake. Ecol Sci. 29(5):456–460.

[CIT0003] Tamura K, Stecher G, Peterson D, Filipski A, Kumar S 2013 MEGA6: molecular evolutionary genetics analysis version 6.0. Mol Biol Evol. 30(12):2725–2729. 24132122 10.1093/molbev/mst197 PMC3840312

[CIT0004] Wolstenholme DR 1992 Animal mitochondrial DNA: structure and evolution. Int Rev Cytol. 141:173–216. 1452431 10.1016/s0074-7696(08)62066-5

[CIT0005] Yang H, Zhang JE, Deng ZX, Luo H, Guo J, He S, Luo M, Zhao B 2016 The complete mitochondrial genome of the golden apple snail *Pomacea canaliculata* (Gastropoda: Ampullariidae). Mitochondrial DNA B. 1(1):45–47. 10.1080/23802359.2015.1137816 PMC7800459 33473402

[CIT0006] Yang H, Zhang JE, Guo J, Deng Z, Luo H, Luo M, Zhao B 2016 The complete mitochondrial genome of the giant African snail *Achatina fulica* (Mollusca: Achatinidae). Mitochondrial DNA A. 27:1622–1624. 10.3109/19401736.2014.958698 25231719

[CIT0007] Yang H, Zhang JE, Luo H, Luo M, Guo J, Deng Z, Zhao B 2016 The complete mitochondrial genome of the mudsnail *Cipangopaludina cathayensis* (Gastropoda: Viviparidae). Mitochondrial DNA A. 27:1892–1894. 10.3109/19401736.2014.971274 25319293

[CIT0008] Zeng T, Yin W, Xia R, Fu C, Jin B 2015 Complete mitochondrial genome of a freshwater snail, *Semisulcospira libertina* (Cerithioidea: Semisulcospiridae). Mitochondrial DNA. 26(6):897–898. 24409867 10.3109/19401736.2013.861449

[CIT0009] Zhou Y 1996 Morphological studies on the reproductive organs and spermatozoa of *Rivularia auriculate* Martens (Gastropoda). Acta Zool Sin. 42(4):343–349.

